# The Immune Adjuvant Effects of Flounder (*Paralichthys olivaceus*) Interleukin-6 on *E. tarda* Subunit Vaccine OmpV

**DOI:** 10.3390/ijms18071445

**Published:** 2017-07-05

**Authors:** Ming Guo, Xiaoqian Tang, Xiuzhen Sheng, Jing Xing, Wenbin Zhan

**Affiliations:** 1 Laboratory of Pathology and Immunology of Aquatic Animals, Ocean University of China, 5 Yushan Road, Qingdao 266003, China; guominghx@163.com (M.G.); tangxq@ouc.edu.cn (X.T.); xzsheng@ouc.edu.cn (X.S.); xingjing@ouc.edu.cn (J.X.); 2 Laboratory for Marine Fisheries Science and Food Production Processes, Qingdao National Laboratory for Marine Science and Technology, No. 1 Wenhai Road, Aoshanwei Town, Jimo, Qingdao 266071, China

**Keywords:** interleukin-6, adjuvant, flounder, *Edwardsiella tarda*, outer membrane protein V, immune response

## Abstract

Interleukin-6 (IL-6) as a pleiotropic cytokine was widely used as an effective adjuvant for vaccines in mammals. In this study, the immune adjuvant effects of two forms of flounder (*Paralichthys olivaceus*) IL-6, including recombinant IL-6 (rIL-6) and pcDNA3.1-IL-6 (pcIL-6), were evaluated and comparatively analyzed on *E. tarda* subunit vaccine recombinant outer membrane protein V (rOmpV). The results showed that the relative percent survivals of flounder vaccinated with rOmpV plus rIL-6 or pcIL-6 were significantly higher than that in the two control groups, rOmpV plus recombinant 6× histidine-tag (rHis) or empty expression vector pcDNA3.1 (pcN3). The levels of specific serum antibodies and surface membrane immunoglobulin-positive (sIg+) lymphocytes in peripheral blood, spleen, and head kidney in the two adjuvant groups were also much higher than that in the two control groups. Compared with the two control groups, higher upregulated expressions of major histocompatibility complex class Iα (*MHCIα*), cluster of differentiation 8α (*CD8α*), *MHCIIα*, *CD4-1*, interleukin-1β (*IL-1β*), and tumor necrosis factor-α (*TNF-α*) were detected in flounder vaccinated with rOmpV plus rIL-6 or pcIL-6 after challenge. In addition, the rOmpV plus rIL-6 could induce significant higher levels of specific serum antibodies, sIg+ lymphocytes and four genes expressions than rOmpV plus pcIL-6. These results demonstrated that both rIL-6 and pcIL-6 used as adjuvants could enhance the immune response and evoke immune protections against *E. tarda* infection, which has a significant value in controlling diseases using vaccines in flounder.

## 1. Introduction

Since vaccines have advantages in safety, environmental friendliness, and long-term efficacy of protection [1], various types of vaccines including attenuated live vaccines, inactivated vaccines, subunit vaccines, and DNA vaccines have been designed for a diverse range of uses in disease prevention in aquaculture [2,3,4]. However, many vaccines such as subunit vaccines fail to induce strong immune responses and obtain satisfactory immune protection when administered without adjuvants. At present, according to the modes of action, adjuvants are generally divided into three types [5,6]. The first group comprises an aluminum hydroxide and oil emulsion adjuvant that functions at the location of the antigen, prolonging antigen exposure and presentation via depot formation [7,8]. The second group refers to inducing a “danger-signal” that provides pathogen associated molecular patterns (PAMPs) of the antigen, recognized by pattern recognition receptors (PRRs) of the host, and activates the host immune responses [9,10]. The third group comprises those specifically targeted to host immune pathways during the antigen recognition process, such as co-stimulation molecular and cytokines [11,12]. Among the adjuvants, the most effective known adjuvants in fish were Freund’s adjuvant and aluminum salts adjuvant. However, due to the various adverse effects observed, such as tissue impairment, necrosis, and weak adjuvanticity, these adjuvants were unsuitable for use in human and animals at a large scale [13,14]. Thus, there is an urgent need for the safety and effectiveness of novel adjuvants in fish vaccines.

Cytokines are a class of highly active multifunctional protein peptide molecules that mediate and regulate immune responses and inflammatory responses, stimulate hematopoietic function, and participate in tissue repair. In mammals, interleukin-6 (IL-6) is a cytokine that has been documented to be involved in many biological functions, including stimulating of T-cell and B-cell growth and differentiation, regulating gene expression, inhibiting cell growth and regulating of acute phase protein synthesis from hepatocytes [15,16,17,18,19]. Moreover, IL-6 in the form of recombinant protein or DNA plasmid has already been used as an efficient adjuvant in various mammalian models. Research has confirmed that mouse IL-6 used as a molecular adjuvant enhanced innate and adaptive immune responses of mouse against the foot-and-mouth disease virus (FMDV) [20]. It was also reported that porcine IL-6 used as a molecular adjuvant promoted antigen-specific and cell-mediated immune responses against Pasteurellosis and Erysipelas suis in a mice model [21]. Moreover, recombinant human IL-6 augmented the tear IgA antibody response of rat against dinitrophenylated pneumococcus (DNP-Pn) [22]. So far, homologues of many mammalian cytokines have been cloned from fish [13,23,24]. Moreover, several cytokines have already been used as efficient adjuvants for co-vaccination with vaccines to gain strong immune responses and ensure highly protective effects against pathogens in fish [25,26]. In teleost, although IL-6 was firstly identified in Japanese pufferfish (*Fugu rubripes*) [27] and then characterized in several other fish species including flounder (*Paralichthys olivaceus*) [28], rainbow trout (*Oncorhynchus mykiss*) [29], sea bream (*Sparus aurata*) [30], Atlantic halibut (*Hippoglossus hippoglossus* L.) [31], and large yellow croaker (*Larimichthys crocea*) [32], the studies on the adjuvant effects of IL-6 are still lacking.

In our previous study, *E. tarda* outer membrane protein OmpV was identified as an efficient subunit vaccine against *E. tarda* in flounder [33]. In this study, the immune adjuvant effects of two forms of flounder IL-6 (rIL-6 and pcIL-6) were evaluated and comparatively analyzed on *E. tarda* subunit vaccine rOmpV in flounder following the vaccination. After vaccination, the production of specific serum antibodies, the percentages of surface membrane immunoglobulin-positive (sIg+) cells in peripheral blood leukocytes (PBL), spleen leukocytes (SL), and head kidney leukocytes (HKL), the expression levels of immune-related genes and the relative percent survival (RPS) were investigated.

## 2. Results

### 2.1. Expression and Purification of Recombinant Interleukin-6 and E. tarda Subunit Vaccine Recombinant Outer Membrant Protein V

**Figure 1:**
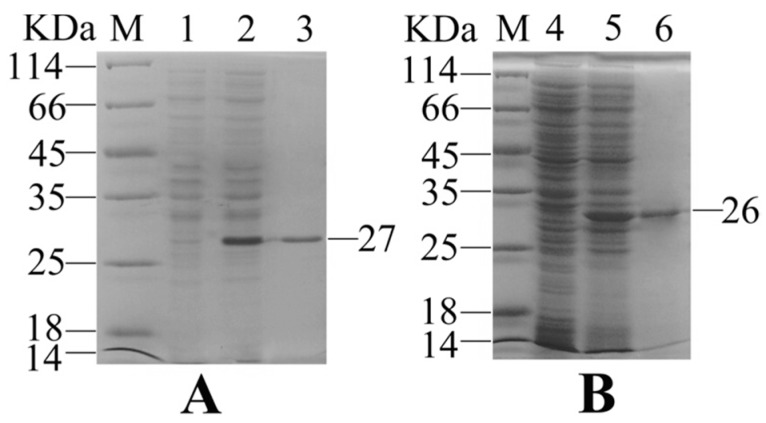
Sodium dodecyl sulfate-polyacrylamide gel electrophoresis (SDS-PAGE) analysis of recombinant interleukin-6 (rIL-6) (**A**) and recombinant outer membrane protein V (rOmpV) (**B**). Lane M: protein marker; Lanes 1and 4: recombinant clones without isopropy-β-d-thiogalactoside (IPTG) induction; Lanes 2 and 5: recombinant clones with IPTG induction; Lanes 3 and 6: purified rIL-6 and rOmpV.

Gene coding OmpV and IL-6 were successfully expressed in *E. coli* BL21 (DE3) with the pET-30a system. Sodium dodecyl sulfate-polyacrylamide gel electrophoresis (SDS-PAGE) analysis showed that the distinct bands of 27 KDa and 26 KDa were matched to the expected molecular masses of rIL-6 and rOmpV, respectively (Figure 1). After purification with the Ni^2+^ affinity chromatography and refolding using a stepwise dialysis method, a high purity of recombinant proteins was obtained.

### 2.2. Tissue Distribution and In Vivo Expression of pcDNA3.1-IL-6 (pcIL-6)

**Figure 2:**
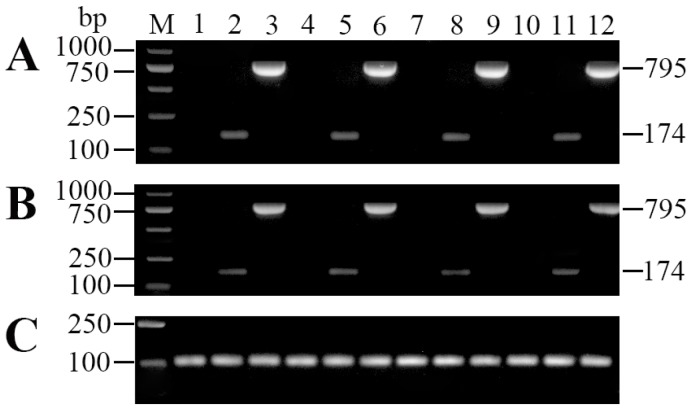
Detection of plasmid pcIL-6 (**A**) and IL-6 transcription (**B**) by polymerase chain reaction (PCR) and reverse transcription polymerase chain reaction (RT-PCR) in vaccinated fish. Flounder were vaccinated with phosphate buffered saline (PBS) (Lanes 1, 4, 7, and 10), rOmpV + pcDNA3.1 (pcN3) (Lanes 2, 5, 8, and 11), and rOmpV + pcDNA3.1-IL-6 (pcIL-6) (Lanes 3, 6, 9 and 12); DNA and RNA were extracted from muscle (Lanes 1, 2, and 3), spleen (Lanes 4, 5, and 6), head kidney (Lanes 7, 8, and 9), and liver (Lanes 10, 11, and 12) at 7 d post-vaccination. The 18S rRNA (**C**) was used as an internal control. Lane M, DNA marker.

Polymerase chain reaction (PCR) and reverse transcription polymerase chain reaction (RT-PCR) analyses were performed to detect the presence of plasmid DNA and IL-6 transcription, respectively. The results showed that both the plasmid DNA and IL-6 transcription were detected in muscle, spleen, head kidney, and liver of rOmpV + pcIL-6 vaccinated fish at 7 d post-vaccination (Figure 2). On the contrary, no plasmids DNA or IL-6 transcription was detected in any sampled tissues of fish vaccinated with phosphate buffered saline (PBS) and rOmpV + pcN3.

### 2.3. Response of Surface Membrane Immunoglobulin-Positive (sIg+) Lymphocytes after Vaccination

**Figure 3:**
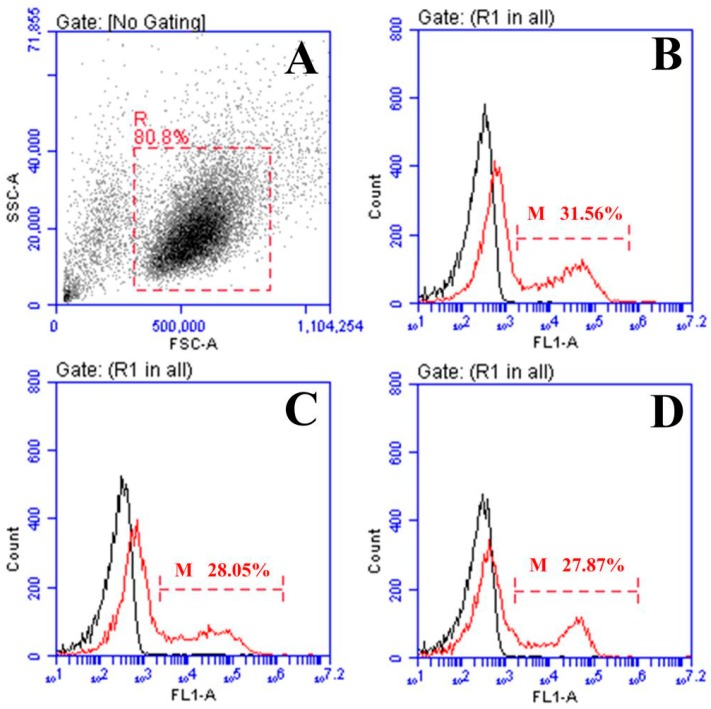
Flow cytometric analysis of sIg+ lymphocytes in flounder vaccinated with rOmpV + pcIL-6 at Week 5 post-vaccination. (**A**) Lymphocytes in peripheral blood were gated (red dash frame, R) on a forward scatter/sideward scatter (FSC / SSC) dot plots. (**B**–**D**) Flounder vaccinated with rOmpV + pcIL-6, combined (smoothed) fluorescein isothiocyanate (FITC) fluorescence histograms of gated lymphocytes (R) showing the percentage of sIg+ lymphocytes (scale of M) in peripheral blood leukocytes (PBL), spleen leukocytes (SL), and head kidney leukocytes (HKL), respectively.

**Figure 4:**
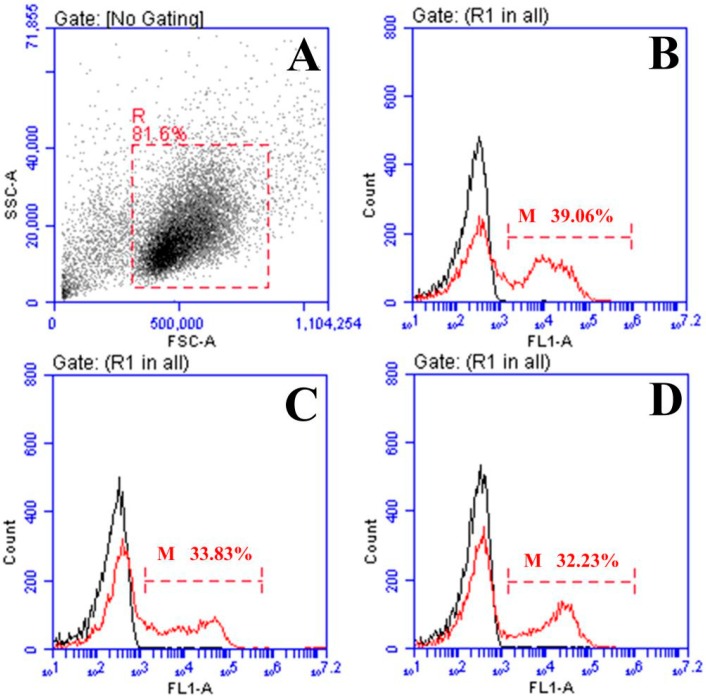
Flow cytometric analysis of sIg+ lymphocytes in flounder vaccinated with rOmpV + rIL-6 at Week 5 post-vaccination. (**A**) FSC/SSC dot plots of lymphocytes in peripheral blood. (**B**–**D**), Flounder vaccinated with rOmpV + rIL-6, combined (smoothed) (FITC) fluorescence histograms of gated lymphocytes (R) showing the percentage of sIg+ lymphocytes (scale of M) in PBL, SL, and HKL, respectively.

**Figure 5:**
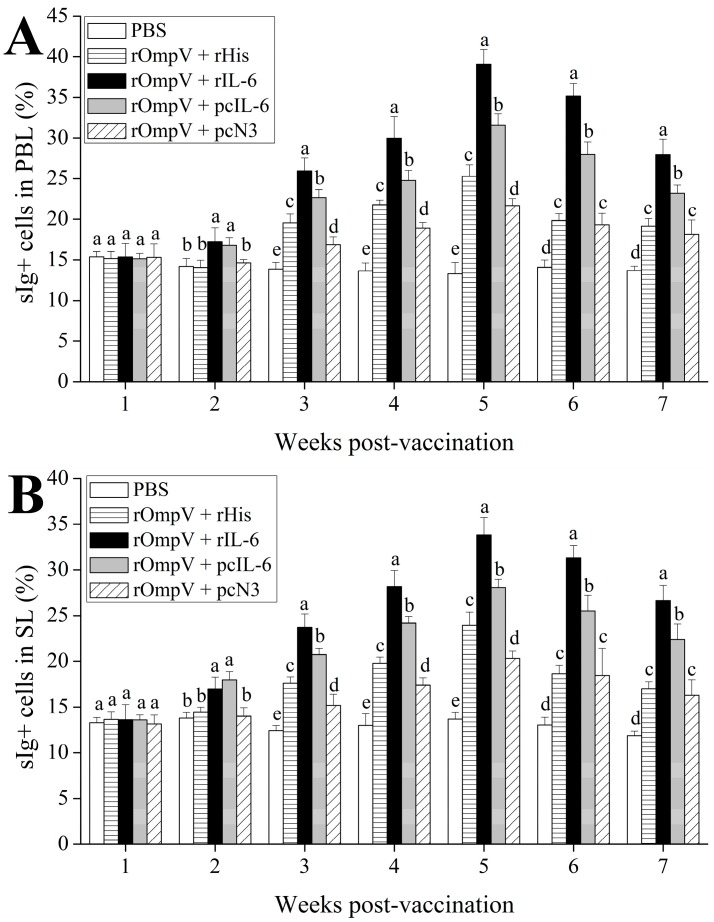

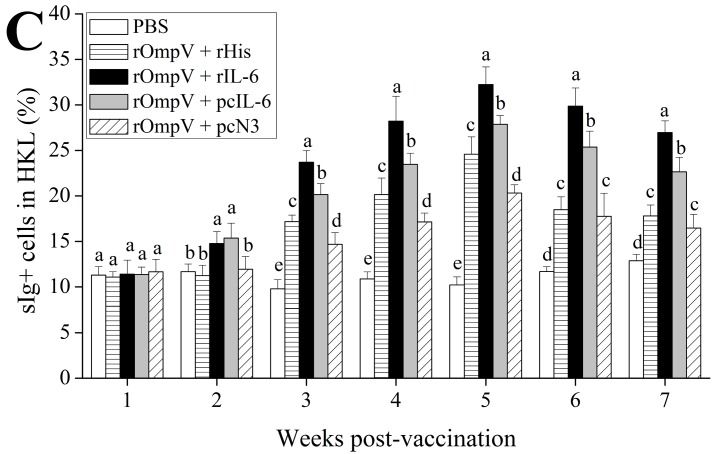
The changes of the levels of sIg+ lymphocytes in PBL (**A**), SL (**B**), and HKL (**C**) of different experimental groups post-vaccination. Data are expressed as the means ± standard error of mean (SEM) (*n* = 3). Different letters above each bar represent the statistical significance (*p* < 0.05) compared to each other at the same time point.

The forward and sideward scatter (FS-SS) dot plot with gated lymphocytes and representing fluorescence histograms of rOmpV + pcIL-6- and rOmpV + rIL-6-vaccinated fish at Week 5 post-vaccination are shown in Figure 3 and Figure 4, respectively, and the variation trends of the percentage of sIg+ lymphocytes in vaccinated fish are summarized in Figure 5. The fluorescence histograms show that the lymphocytes unstained with mAb 2D8 only have a single peak (black line), which was used for negative control. The lymphocytes stained with mAb 2D8 exhibit two peaks on fluorescence histograms (red line), and the second peak (M) represent the subpopulation of sIg+ lymphocytes in PBL, SL, and HKL. In the blank control group, the levels of sIg+ lymphocytes in PBL, SL, and HKL maintained a steady level during the experimental period, whereas the levels of sIg+ lymphocytes in the three lymphoid organs of the four rOmpV vaccination groups gradually increased and reached their peak levels at Week 5 post-vaccination, then descended slowly. Compared to the two control groups, the levels of sIg+ lymphocytes in PBL, SL, and HKL of the two adjuvant groups showed a much quicker and stronger response since the second week post-vaccination (*p* < 0.05). Between the two adjuvant groups, higher levels of sIg+ lymphocytes in PBL, SL and HKL were detected in the rOmpV + rIL-6 group compared with the rOmpV + pcIL-6 group at Week 3 to Week 7 post-vaccination (*p* < 0.05). In addition, in the two control groups, the group of rOmpV + rHis induced higher levels of sIg+ lymphocytes in PBL, SL, and HKL compared with the rOmpV + pcN3 group at Week 3 to Week 5 post vaccination (*p* < 0.05).

**Figure 6:**
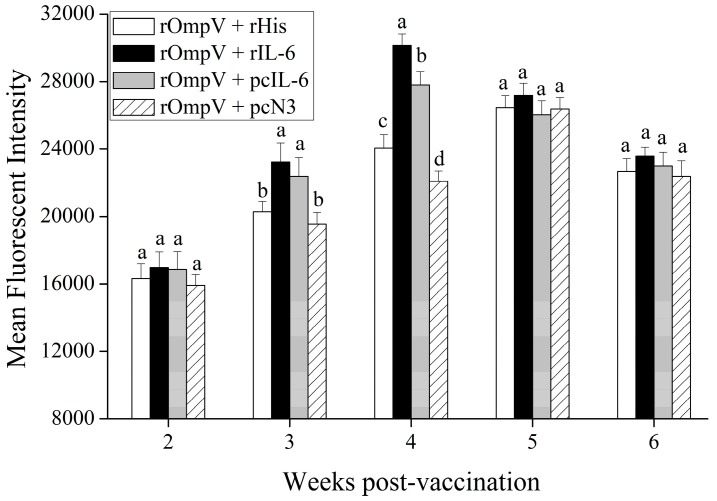
The changes of mean fluorescence intensity of sIg+ lymphocytes in head kidney of vaccinated flounder. Data are expressed as the means ± SEM (*n* = 3). Different letters above each bar represent the statistical significance (*p* < 0.05) compared to each other at the same time point.

We also analyzed the mean fluorescence intensity (MFI) of sIg+ lymphocytes in head kidney of flounder in the four vaccination groups post-vaccination. The results showed that the MFI of sIg+ lymphocytes in each experimental group significantly increased, and reached their peaks at Week 4 or Week 5 post vaccination, then declined. Compared with the two control groups, higher levels of the MFI of sIg+ lymphocytes were observed in the two adjuvant groups at Week 3 and Week 4 post-vaccination (*p* < 0.05). Moreover, the MFI of sIg+ lymphocytes in the two adjuvant groups reached the peak levels at Week 4 post-vaccination, which was earlier than those in the control groups at Week 5 post-vaccination, respectively. Furthermore, in the two adjuvant groups, higher levels of the MFI of sIg+ lymphocytes were found in the rOmpV + rIL-6 group compared with the rOmpV + pcIL-6 group (*p* < 0.05) at Week 4 post vaccination. In addition, the group of rOmpV + rHis induced a higher level of the MFI of sIg+ lymphocytes than that in the rOmpV + pcIL-6 group at Week 4 post vaccination (*p* < 0.05) (Figure 6).

### 2.4. Response of Specific Serum Antibodies

**Figure 7:**
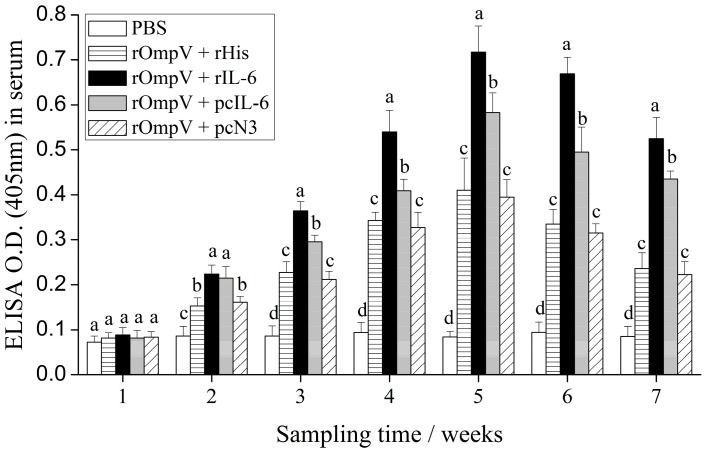
Serum antibodies detection in vaccinated fish by enzyme-linked immunosorbent assay (ELISA)*.* Serum was randomly collected from three fish at Weeks 1, 2, 3, 4, 5, 6, and 7 post-vaccination. Results are expressed as the means ± SEM (*n* = 3). Different letters on the bars represent the statistical significance (*p* < 0.05).

The specific serum antibodies of vaccinated flounder in each experimental group were detected by enzyme-linked immunosorbent assay (ELISA) from Weeks 1 to 7 post-vaccination (Figure 7). Compared to the blank control group, the levels of specific serum antibodies in the four rOmpV vaccination groups showed the same dynamics trend. The levels gradually increased and reached their peak levels at Week 5 post-vaccination, then descended slowly. Two weeks post-vaccination, the specific serum antibodies levels of the two adjuvant groups were significantly higher than the two control groups, respectively (*p* < 0.05). Between the two adjuvant groups, the levels of specific serum antibodies in the rOmpV + rIL-6 group were significantly higher than the rOmpV + pcIL-6 group at Week 3 to Week 7 post-vaccination (*p* < 0.05). However, the levels of specific serum antibodies between the two control groups showed no significant difference at all detected time points (*p* > 0.05).

### 2.5. Expression of Immune-Related Genes

**Figure 8:**
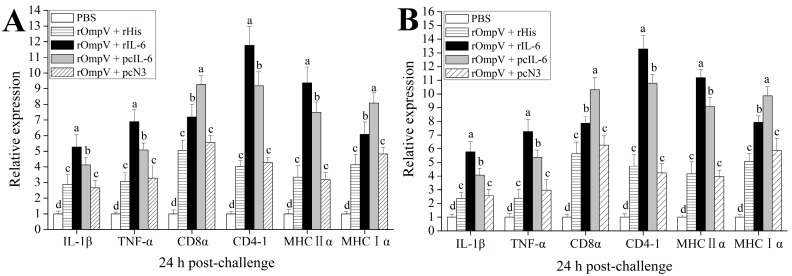
qRT-PCR analysis of the expression of immune-related genes in spleen (**A**) and head kidney (**B**) of vaccinated fish at 24 h post-challenge. The expression of each gene in spleen and head kidney was normalized to that of 18S rRNA and the mRNA level of the PBS group was set as one. The results are presented as the mean ± SEM of three fish. Different letters on the bars indicated the significance difference (*p* < 0.05).

The expressions of *CD4-1*, *MHCIIα*, *CD8α*, *MHCIα*, *TNF-α*, and *IL-1β* in spleen and head kidney of vaccinated flounder were investigated by qRT-PCR at 24 h post-challenge. The results showed that the mRNA levels of all investigated genes were induced significantly in the four rOmpV vaccination groups compared with the blank control group (Figure 8). Compared to the two control groups, higher mRNA levels of all investigated genes were detected in the two adjuvant groups (*p* < 0.05). Moreover, the mRNA levels of *MHCIIα*, *CD4-1*, *IL-1β*, and *TNF-α* in the rOmpV + rIL-6 group were much higher than that in the rOmpV + pcIL-6 group (*p* < 0.05), while the mRNA levels of *MHCIα* and *CD8α* in the rOmpV + pcIL-6 group were much higher than that in the rOmpV + rIL-6 group (*p* < 0.05). However, there was no significant difference of the mRNA levels of all investigated genes between the two control groups (*p* > 0.05).

### 2.6. Protection against E. tarda Infection

**Figure 9:**
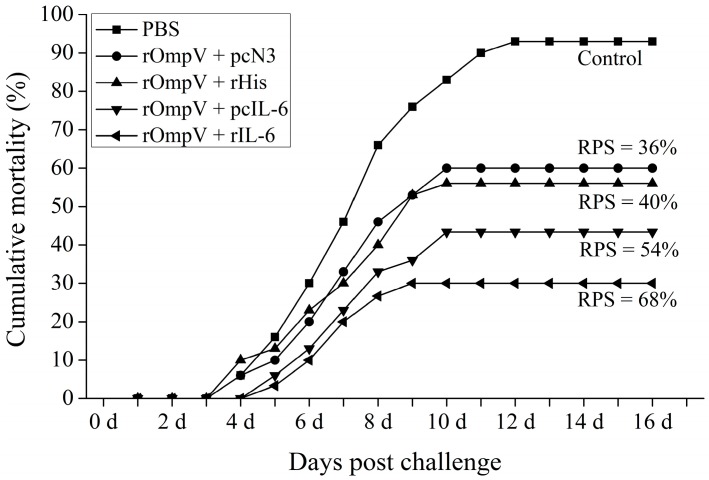
Cumulative mortality rates of vaccinated flounder after challenge with live *E. tarda* at Week 5 post-vaccination.

The cumulative mortality rates of vaccinated fish after challenging with live *E. tarda* at five weeks post-vaccination are shown in Figure 9. In the blank control group, the challenged fish began to die at Day 3, then increased rapidly at one to two weeks and the cumulative mortality finally reached 93% at Day 12. However, a lower cumulative mortality rate was observed in the four rOmpV vaccination groups. The cumulative mortality rate of the rOmpV + pcN3, rOmpV + pcIL-6, rOmpV + rHis and rOmpV + rIL-6 group was 60%, 43%, 56%, and 30%, respectively. Hence, the RPS of rOmpV + rIL-6 and rOmpV + pcIL-6 reached 68% and 54%, respectively, which was higher than that in the two control groups, rOmpV plus rHis (40%) or pcN3 (36%). Some typical clinical signs of edwarsiellosis including ascites and ulcer were observed in infected flounder. Bacteria tests on the infected flounder also demonstrated that *E. tarda* was the only type of pathogen that caused the death of flounder.

## 3. Discussion

Previous studies on functional properties of IL-6 in mammals showed that it had an application prospect as an efficient adjuvant. In teleost, although several IL-6 have been cloned and characterized, the study on whether they have an adjuvant effect has not been carried out. The present work focuses on the effects of two forms of IL-6 (pcIL-6 and rIL-6) as a vaccine adjuvant on the responses of the specific serum antibodies, the sIg+ lymphocytes in three lymphoid organs and the expression of immune-related genes of flounder, and the immunoprotective efficacies of all experimental groups were also measured. The results showed that both rIL-6 and pcIL-6 used as adjuvants enhanced the immune responses, including cellular, humoral and inflammatory immunity, and evoked highly protective effects against *E. tarda* infection. Therefore, our research demonstrated that the IL-6 could be used as an effective immune adjuvant to promote comprehensive immunity of flounder.

IL-6 was first defined as a B-cell differentiation factor (BSF-2) in antigen-stimulated peripheral blood mononuclear cells that induce immunoglobulin (Ig) production [34]. Currently, the pathways of IL-6 activating Ig production have been demonstrated in mammals. IL-6, IL-6R and dimerization of glycoprotein-130 (gp130) form a complex that activates the Janus kinase (JAK) and signal transducers and activators of transcription (STAT) pathway, and ultimately, the STAT3 pathway is activated by IL-6 to induce Ig production [16,35]. Recently, in teleost, it was reported that recombinant trout IL-6 could activate the STAT3 pathway in trout macrophages [36]. Consistent with the findings of this study, the STAT3 pathway was activated by fugu IL-6 via gp130 and IL-6R and increased the production of antibodies secretion [37]. Another study in trout also demonstrated that recombinant IL-6 of trout could induce the proliferation and differentiation of unstimulated B-cells, and augment the secretion of IgM [38]. In the present study, rOmpV + pcIL-6 and rOmpV + rIL-6 induced higher levels of sIg+ lymphocytes and higher production of specific serum antibodies against *E. tarda* compared with the two control groups, and higher immune protections were also found in rOmpV + pcIL-6 and rOmpV + rIL-6 groups. These results demonstrated that both pcIL-6 and rIL-6 enhanced the humoral immune responses and evoked the immune protection against *E. tarda*. Similarly, previous studies have also shown that IL-6 in the form of recombinant protein or plasmid DNA used as adjuvant could enhance antibody-mediated immune responses and evoke highly protective effects [21,39,40].

After antigen stimulation, naive B-cells undergo several stages of differentiation, including mature B-cells, activated B-cells, plasmablasts, and eventually differentiate into plasma cells [41,42]. It was reported that mature B-cells have high expression of surface Ig (sIg), activated B-cells and plasmablasts have lower expression of sIg, and plasma cells lack the sIg in the process of B-cell differentiation [41,43]. Previous studies also revealed that head kidney contains large numbers of plasmablasts and plasma cells, which could effectively proliferate after immunization [44]. Therefore, we analyzed the changes of the MFI of sIg+ lymphocytes in head kidney to evaluate the differentiation process of B-cells after immunization in the present study. We found that the MFI of sIg+ lymphocytes in the four rOmpV vaccination groups gradually increased post-vaccination, reached their peaks, then underwent a slight decline. These results suggested that the early rise of the MFI of sIg+ lymphocytes was due to the proliferation and maturation of B-cells and that the later decrease was due to the differentiation of mature B-cells into the plasma cells. Moreover, the MFIs of sIg+ lymphocytes in the two adjuvant groups were much higher and reached the peak levels earlier compared with the two control groups. These results suggested that pcIL-6 or rIL-6 could promote the proliferation and differentiation of B-cells. Similarly, previous studies in mammals also found that IL-6 had the ability to induce B-cells proliferation and differentiation [17].

It was noted that IL-6 could induce B-cells to produce immunoglobulins and also played an important role in the T cell-mediated immune responses. Studies in mice showed that purified IL-6 could stimulate mature thymic and peripheral T-cell proliferation and enhance the differentiation of mouse cytolytic T-cell precursors in lymphocyte culture mixed primary allogeneic lymphocyte culture [45]. Similar studies also demonstrated that IL-6 could induce the differentiation of thymocytes and splenic cytotoxic T-lymphocytes (CTL) in the presence of IL-2 in a human and mice model [46,47]. In the present study, the mRNA levels of *MHCIα*, *CD8α*, *MHCIIα*, and *CD4-1* were significantly higher in spleen and head kidney of the rOmpV + rIL-6 and the rOmpV + pcIL-6 groups than that in the two control groups, respectively. These results demonstrated that both pcIL-6 and rIL-6 boosted the T cell-mediated immune responses of flounder. Previous study in mice also showed that IL-6 used as molecular adjuvant enhanced T-cell mediated immune responses induced by VP1 DNA vaccine [20]. In this study, the mRNA levels of *IL-1β* and *TNF-α* were also significantly induced by rOmpV + rIL-6- and rOmpV + pcIL-6-vaccinated fish compared with the two control groups respectively, which indicated that both rIL-6 and pcIL-6 strengthened the inflammatory immune responses. Similarly, flounder *IL-1β* and channel catfish *IL-8* used as adjuvants also significantly enhanced the mRNA levels of *IL-1β* and *TNF-α* in a flounder and channel catfish model [25,26].

Recombinant cytokines play roles in the form of mature proteins in vivo, while plasmid cytokines need to be expressed and synthesized by the transcription and translation mechanisms of the immunized animals to produce the corresponding cytokines proteins [48,49]. Thus, we explored whether different forms of IL-6 as potential adjuvant candidates could elicit different immune responses under the same vaccination conditions. In this work, rOmpV + rIL-6-vaccinated fish induced higher levels of sIg+ lymphocytes and specific serum antibodies compared with rOmpV + pcIL-6-vaccinated fish, and higher immune protection was also found. These results showed that the humoral immunity enhanced by rIL-6 was much higher in magnitude than that of pcIL-6, suggesting that the stronger humoral immune response was related to higher protective effects. Moreover, *IL-1β* and *TNF-α* are important pro-inflammatory cytokines that function in the recruitment and activation of macrophages, and in the stimulation of the adaptive immune response [50]. In this work, rOmpV + rIL-6 vaccinated fish also induced higher mRNA levels of *IL-1β* and *TNF-α* compared with rOmpV + pcIL-6 vaccinated fish, which indicated that the inflammatory immunity enhanced by rIL-6 was higher in magnitude than that of pcIL-6. MHCII is responsible for binding antigen peptides derived from exogenous antigen for activating helper CD4+ T cell-mediated humoral immunity [51]. In this study, rOmpV + rIL-6 evoked higher mRNA levels of *MHCIIα* and *CD4-1* compared with rOmpV + pcIL-6 in the vaccinated fish, suggesting that the MHCII antigen presentation was much more activated by rIL-6. However, the mRNA levels of *MHCIα* and *CD8α* in the rOmpV + pcIL-6 vaccinated flounder were much higher than that in rOmpV + rIL-6 vaccinated fish. MHCI is responsible for binding antigen peptides derived from endogenous antigen for initiating cytotoxic CD8+ T cell-mediated cellular immunity [23,51]. This result suggested that pcIL-6 activated stronger cellular immunity than rIL-6. The expressions differences of immune-related genes between two adjuvant groups might be due to the different properties of the two forms of IL-6 and the different concentrations of effective IL-6 derived from rIL-6 and pcIL-6.

## 4. Materials and Methods

### 4.1. Expression, Purification, and Refolding of Recombinant Interleukin-6

The truncate *IL-6* gene excluding the region coding for its signal peptide was amplified using specific primers IL-6-F and IL-6-R (Table 1). The purified PCR products of IL-6 was cloned into the pET-30a expression vector (Novagen, Merck Millipore, Darmstadt, Gemany) to constructed recombinant clones using *Bam*HI and *Hin*dIII restriction enzymes. For expression, the recombinant clones were transformed into chemically-competent *E. coli* BL21 (DE3) and grown in Luria-Bertani (LB) at 37 °C to mid-logarithmic phase. When the OD_600_ of the cultures reached 0.6, 1 mM isopropyl thiogalactoside (IPTG) was added and grown at 37 °C for an additional 10 h. Then, the cultures were centrifuged, and the His-tagged rIL-6 was purified using His Trap^TM^ HP Ni-Agarose (GE healthcare, Beijing, China). For refolding, the purification of the protein rIL-6 was conducted by the stepwise dialysis method [52,53,54], from containing 6, 3, 2, 1, 0.5, 0 M urea of guanidine HCl solutions to phosphate-buffered saline (PBS), and oxidized glutathione (GSSG) and l-arginine were added at the state of containing 1 and 0.5 M urea of guanidine HCl dialysis fluid. Then, the purified and refolded protein was treated with Triton X-114 to remove endotoxin [55] and analyzed by SDS-PAGE. The concentrations of proteins were quantified using the Bradford method.

### 4.2. Construction of the pcIL-6 Plasmid

Based on the region coding for the *IL-6* gene and Kozak consensus sequence [56,57,58], the specific primers pcN3-IL-6-F/pcN3-IL-6-R (Table 1) were designed. The expression vector pcDNA3.1 (pcN3) was purchased from a commercial company (Invitrogen, Carlsbad, CA, USA). The PCR products of pcN3-IL-6 and the pcN3 vector were both digested with *Bam*HI and *Hin*dIII restriction enzymes, and then ligation by T_4_ ligase overnight. The linked product was then transformed into Trans5α chemically competent cells (Transgen, Beijing, China) and grown in LB broth at 37 °C for 1 h. Then, the suspended cells were centrifuged and inoculated in LB plate with ampicillin (100 μg/mL) at 37 °C for 15 h. Subsequently, the transformants were screened and sequenced to confirm the target gene. The correct constructed recombinant plasmid was named pcDNA3.1-IL-6 (pcIL-6). For the purpose of vaccination, the recombinant plasmid pcIL-6 was treated with an EndoFree Plasmid Kit (Tiangen, Beijing, China) to remove the endotoxin.

### 4.3. Expression, Purification, and Refolding of Recombinant OmpV

The specific primers OmpV-F/OmpV-R (Table 1) were designed to amplify the open reading frames (ORF), excluding signal peptides according to the genome sequence encoding OmpV gene (GenBank No. ETAE_2675). The expression, purification and refolding procedure for the OmpV gene was described as above.

### 4.4. Vaccination

Six hundred healthy flounders of approximately 15 to 17 cm (~30 g) were obtained from a fish farm in Shandong Province, China. The fish were reared in tanks containing aerated sand-filtered seawater at 21 ± 1 °C for two weeks before vaccination and fed daily with commercial diet.

Flounders were randomly divided into five groups (1, 2, 3, 4, and 5), 120 fish per group. The specific immunization program is shown in Table 2; the fish of two adjuvant groups were intramuscularly injected with 100 μL PBS containing 200 μg rOmpV plus 20 μg rIL-6 or pcIL-6; the fish injected with 100 μL PBS containing 200 μg rOmpV plus 20 μg rHis or pcN3 were the controls respectively; and the fish only injected with 100 μL PBS were set as the blank control. The rHis was the recombinant protein form of the 6 × histidine-tag purified from the pET-30a express system. The molecular mass of rHis was approximately 8 KDa, which was purchased from a commercial company (APExBIO, Glendale, CA, USA).

### 4.5. Sampling

The serum and the leucocytes of peripheral blood (PBL), spleen (SL) and head kidney (HKL) were randomly sampled from three fish in each experimental group at Weeks 1, 2, 3, 4, 5, 6, and 7 post-vaccination. For serum isolation, blood was collected from the venipuncture and allowed to clot for overnight at 4 °C. The serum was obtained by centrifugation at 5000× *g* for 10 min and stored at −20 °C until use. For qRT-PCR, the RNA was extracted from the total tissues of spleen and head kidney of three random fish in each experimental group at 24 h post-challenge. For the detection of plasmid DNA and IL-6 transcripts, muscle, head kidney, spleen, and liver were sampled from fish vaccinated with rOmpV + pcN3, rOmpV + pcIL-6 and PBS at 7 d post-vaccination. Tissue samples were kept in the Sample Protector (Baosheng, Dalian, China) and stored at −20 °C until usage. Fish were anaesthetized with MS-222 prior to sampling. The fish used in this study was carried out strictly in line with procedures in the Guide for the Use of Experimental Animals of the Ocean University of China. In this study, the methods used in the animal experiments were approved by the Instructional Animal Care and Use Committee of the Ocean University of China (permit number: 20150101). All possible effort was dedicated to minimizing suffering.

### 4.6. Distribution and Expression of pcIL-6 in Fish Tissues

Total DNA and RNA were extracted from muscle, spleen, head kidney, and liver at 7 day post-vaccination using the TIANamp DNA kit (Tiangen, Beijing, China) and RNAiso (Baosheng, Dalian, China), respectively, according to the manufacture instruction. Two micrograms of total DNA were quantified using a NanoDrop-8000 spectrophotometer (Thermo Scientific, Waltham, MA, USA), then diluted 10-fold in distilled water and used as templates for PCR analysis to detect the distribution of pcIL-6 in sampled tissues of flounder. Then, one microgram of total RNA was quantified and used for cDNA synthesis by PrimeScript^TM^ RT-PCR Kit (Baosheng, Dalian, China) following the manufacture’s protocol. The kit contains a reagent named gDNA for removal of the genomic DNA. After that, the cDNA samples were diluted 10-fold in distilled water and used as templates for the detection of the transcription of pcIL-6. RT-PCR were performed as described previously [59] using 18S rRNA as an internal control. The primers pcDNAG-F/pcDNAG-R used for PCR and RT-PCR are specific to pcN3-IL-6 and listed in Table 1.

### 4.7. Flow Cytometric Immunofluorescence Analysis

The leucocytes of peripheral blood, spleen, and head kidney in each experimental group were isolated according to the procedure developed by our previous study [60]. The density of isolated PBL, SL, and HKL of vaccinated flounder was diluted to 10^6^ cells mL^−1^ in PBS, and then incubated with mAb 2D8 (1:1000 diluted in PBS), which was previously produced by our laboratory [61]. After incubation at 37 °C for 1 h, the PBL, SL, and HKL were washed thrice with PBS containing 5% (*v*/*v*) newborn calf serum, then incubated with goat-anti-mouse Ig-FITC (1:256 diluted in PBS, Sigma-Aldrich, St. Louis, MO, USA) at 37 °C for 45 min. After being washed again as above, suspensions of PBL, SL, and HKL were analyzed by Accuri C6 cytometer (BD, Accuri^TM^, Piscataway, NJ, USA).

### 4.8. Detection of the Serum Antibodies against E. tarda by Enzyme-Linked Immunosorbent Assay

Specific serum antibody detection was determined by ELISA according to previous studies [62]. Briefly, each well of flat-bottom microplates was covered with 100 μL diluted *E. tarda* (1 × 10^8^ CFU mL^−^^1^) overnight at 4 °C. After washing with phosphate buffered saline tween (PBST) and blocking with 3% BSA in PBS for 1 h at 37 °C, the serum (1:20 diluted in PBS) collected from different experimental groups was added 100 μL per well in triplicate and then incubated for 2 h at 37 °C. The secondary and third antibody were mAb 2D8 (1:1000 diluted in PBS) and goat-anti-mouse Ig-alkaline phosphatase conjugate (1:5000 in PBS, Sigma-Aldrich, St. Louis, MO, USA), which were added 100 μL per well and incubated at 37 °C for 1 h, respectively. After the last washing, 100 μL of 0.1% (*w*/*v*) *p*-nitrophenyl phosphate (pNPP, Sigma) in 50 mM carbonate-bicarbonate buffer (pH 9.8) containing 0.5 mM MgCl_2_ were added to each well and incubated for 30 min at room temperature in the dark. The reaction was stopped with 50 μL per well of 2 M NaOH, and absorbance was measured with an automatic ELISA reader at 405 nm.

### 4.9. Analysis of the Expression of Immune-Related Genes by qRT-PCR

Spleen and head kidney were randomly sampled from three fish in each experimental group at 24 h after challenge. Total RNA extraction and cDNA synthesis were carried out as described above. qRT-PCR was carried out with SYBR Green I Master Mix (Roche, Basel, Switzerland) in a LightCycle^®^ 480 II Real-Time PCR System (Roche, Switzerland). Each assay was performed in triplicate with 18S rRNA as the internal control. The primers of immune-related genes including *MHCIα*, *MHCIIα*, *CD4-1*, *CD8α*, *IL-1β* and *TNF-α* gene are all listed in Table 1. All of the data were analyzed using the 2^−^^ΔΔ*C*t^ method [63].

### 4.10. Challenge

Thirty fish were randomly selected from each experimental group for challenge at Week 5 post-vaccination. The *E. tarda* HC01090721 used for the challenge was cultured in BHI broth at 30 °C for 24 h [60]. The fish was intraperitoneal injected with a dose of 100 μL per fish containing 1 × 10^7^ CFU mL^−^^1^ live *E. tarda*. Mortalities were monitored over a period of 20 days after the challenge, and the relative percent survival rate (RPS) was calculated as previously described [64].

### 4.11. Statistical Analysis

All of the statistical analyses were carried out with SPSS 19.0 software (SPSS Inc., IBM, Armonk, NY, USA). The differences were determined using a one-way analysis of variance (ANOVA). In all cases, the results were expression as the means ± SD (standard deviation), and the significance level was defined as *p* < 0.05.

## 5. Conclusions

This study showed that IL-6 in the form of recombinant or plasmid DNA (rIL-6 or pcIL-6) used as an adjuvant can enhance the immune responses including cellular, humoral, and inflammatory immunity and evoked higher immune protection rates against *E. tarda* infection. Compared with pcIL-6, rIL-6 can induce much stronger humoral and inflammatory immune responses, whereas the cellular immunity enhanced by pcIL-6 was much stronger than rIL-6. The resultant data suggested that IL-6 of flounder could be employed as an effective immune adjuvant.

## Figures and Tables

**Table 1:** Primers used in this study.

No.	Primer Name	Primer Sequence (5′→3′) ^a^	Source
1	IL-6-F	CGGGATCCGCTCCAGTCGAATACGAGCCCAC (*Bam*HI)	DQ267937.1
2	IL-6-R	CCCAAGCTTTGTCATTTGGTAAGAGGGATGGA (*Hin*dII)	
3	18sRNA-F	GGTCTGTGATGCCCTTAGATGTC	EF126037
4	18sRNA-R	AGTGGGGTTCAGCGGGTTAC	
5	pcN3-IL-6-F	CCCAAGCTTACCATGGCTCCAGTCGAATACGA (*Hin*dIIII)	DQ267937.1
6	pcN3-IL-6-R	CGGGATCCTAATGTCATTTGGTAAGAGGGAT (*Bam*HI)	
7	pcDNAG-F	TAATACGACTCACTATAGGG	Invitrogen
8	pcDNAG-R	TAGAAGGCACAGTCGAGG	
9	OmpV-F	CGGGATCCGAGGGCCAGACGGTTTCTCTG (*Bam*HI)	ETAE 1239
10	OmpV-R	CCAAGCTTGAAGCGGTAGCCGACACCCAGG (*Hin*dII)	
11	IL-1β-F	CTGTCGTTCTGGGCATCAAA	AB720983
12	IL-1β-R	AACAGAAATCGCACCATCTCACT	
13	TNFα-F	GTCCTGGCGTTTTCTTGGTA	AB040448
14	TNFα-R	CTTGGCTCTGCTGCTGATTT	
15	MHCIα-F	AGACCACAGGCTGTTATCACCA	AB126921
16	MHCIα-R	TCTTCCCATGCTCCACGAA	
17	MHCIIα-F	ACAGGGACGGAACTTATCAACG	AY997530
18	MHCIIα-R	TCATCGGACTGGAGGGAGG	
19	CD4-1-F	CCAGTGGTCCCCACCTAAAA	AB643634
20	CD4-1-R	CACTTCTGGGACGGTGAGATG	
21	CD8α-F	CCTCTCCCCATACATTGATTCC	AB082957
22	CD8α-R	CCGAGCTTTGCTGAAGGACTT	

^a^ the underline letters represent the restriction enzyme sites.

**Table 2:** Groups of experimental fish.

Group	Treatment
1	100 μL PBS
2	200 μg rOmpV + 20 μg rHis
3	200 μg rOmpV + 20 μg rIL-6
4	200 μg rOmpV + 20 μg pcN3
5	200 μg rOmpV + 20 μg pcIL-6
